# Impact of the COVID-19 Pandemic on Primate Research and Conservation

**DOI:** 10.3390/ani12091214

**Published:** 2022-05-08

**Authors:** Kim E. Reuter, Seheno Andriantsaralaza, Malene Friis Hansen, Marni LaFleur, Leandro Jerusalinsky, Edward E. Louis, Jonah Ratzimbazafy, Elizabeth A. Williamson, Russell A. Mittermeier

**Affiliations:** 1 Lemur Love, San Diego, CA 92122, USA; 2 College of Arts and Sciences, University of San Diego, San Diego, CA 92093, USA; 3 IUCN SSC Primate Specialist Group Executive Committee, c/o Re:wild, Austin, TX 78767, USA; 4 The Long-Tailed Macaque Project, 5000 Copenhagen, Denmark; 5 Behavioural Ecology Group, Department of Biology, University of Copenhagen, 2100 Copenhagen, Denmark; 6 School of Social Sciences, Oxford Brookes University, Oxford OX3 0PB, UK; 7 Centro Nacional de Pesquisa e Conservação de Primatas Brasileiros, Instituto Chico Mendes de Conservação da Biodiversidade (ICMBio/CPB), Cabedelo 58010-480, Brazil; 8 Center for Conservation and Research, Omaha’s Henry Doorly Zoo and Aquarium, Omaha, NE 68107, USA; 9 Groupe D’étude et de Recherche Sur Les Primates (GERP), Antananarivo 101, Madagascar; 10 Faculty of Natural Sciences, University of Stirling, Stirling FK9 4LA, UK; 11 Re:wild, Austin, TX 78767, USA

**Keywords:** primates, sustainability, conservation, novel coronavirus, SARS-CoV-2, COVID-19

## Abstract

**Simple Summary:**

The Coronavirus Disease 2019 (COVID-19) pandemic has made it harder to effectively protect and manage biodiversity, and this could make it more difficult for countries to show progress towards the Sustainable Development Goals (SDGs). Here, we surveyed experts in early 2022 from 30 countries to collect data on the impacts of COVID-19 on non-human primate research and conservation initiatives. Of the 93 experts that responded to our survey, we found that 39% had not been able to visit any of their field sites since March 2020 and only one out of ten had managed to achieve at least 76–100% of their planned primate-related work since March 2020. Six out of ten respondents (61%) felt that primate conservation efforts in protected areas were worse than before the onset of the COVID-19 pandemic and one-third (33%) felt hunting was happening more frequently than before. This study provides evidence of the impacts of COVID-19 on progress towards achieving SDG15 (Life on Land) and provides practical lessons learned for biodiversity conservation efforts moving forward.

**Abstract:**

There is evidence to suggest that the Coronavirus Disease 2019 (COVID-19) pandemic may hamper our achievement of the Sustainable Development Goals (SDGs). Here, we use non-human primates as a case study to examine the impacts of COVID-19 on the ability to achieve biodiversity conservation and management sustainability targets. We collected data through a survey of members of the IUCN SSC Primate Specialist Group from January to March 2022. Of the 93 experts that responded to our survey, we found that 39% had not been able to visit any of their field sites since March 2020, 54% said they had less funding available for their primate-related work, and only one out of ten said they had managed to achieve at least 76–100% of their planned primate-related work since March 2020. Six out of ten respondents (61%) felt that primate conservation efforts in protected areas were worse than before the onset of the COVID-19 pandemic and one-third (33%) felt hunting was happening more frequently than before. This study provides evidence of the impacts of COVID-19 on progress towards achieving the SDGs, and provides practical lessons learned for biodiversity conservation efforts moving forward.

## 1. Introduction

As one of the deadliest diseases to emerge in the 21st century, the Coronavirus Disease 2019 (COVID-19) pandemic continues to impact global economies on an unprecedented scale. With an initial impact described by the World Bank as causing the largest economic decline since World War II [1], the first year of the pandemic sparked large-scale societal shifts, such as the mass exodus of millions of urban laborers to rural parts of India [2], a 4.5% increase in sovereign debt levels across sub-Saharan Africa [3], and one-in-four employed people in the United Kingdom being furloughed (temporarily suspended from work duties, with the government paying their partial salaries) by their employers [4]. To counteract these impacts, governments invested billions to kickstart their economies; the World Bank alone invested over USD 157 billion across a 15-month period, which was 60% more than it invested in the 15-month period prior to the pandemic [5]. This economic recovery effort is described by many governments and stakeholders as an opportunity to move away from business-as-usual and to reinvigorate a drive towards sustainability and meeting the Sustainable Development Goals [6]. The reality, however, is that the COVID-19 recovery has been more ‘brown’ (i.e., unsustainable business-as-usual) than ‘green’ (i.e., environmentally sustainable) [6].

The COVID-19 pandemic has also impacted global biodiversity (see for example, [7,8]). Prior to the onset of COVID-19, the indirect economic drivers of environmental degradation were already well documented (e.g., [9]), and included loss of biodiversity, decreased functionality of ecosystems, and landscape degradation. Following the onset of the pandemic, and although global carbon dioxide emissions fell by 6.4% (2.3 billion tonnes) in 2020 (primarily due to restrictions on travel [10]), there have been reports of increases in forest loss [11], pollution from plastic medical waste in the ocean [12], and supply chain disruptions that impacted biodiversity in unexpected ways [13]. People have also changed the way they interact with the nature around them during the COVID-19 pandemic, including their increased use of urban parks and green spaces [14] and consumption of wildlife [15]. The combined effects of these changes in how humans interact with and use biodiversity, are not yet known.

The COVID-19 pandemic has made it more difficult to protect and manage biodiversity, including in and around protected areas. Several studies have documented the impact of lockdowns and national and international travel restrictions on the ability to conduct routine monitoring activities (e.g., [16,17,18,19]). In some cases, the interruption of funding flows and regular tourism activities have negatively impacted the functioning of day-to-day protected area management [18,20]. In Madagascar, for example, the COVID-19 pandemic resulted in the reduction in salaries for staff and local personnel (including local park rangers) and a move to remote protected area management by teleworking and phone-based communications with rangers [19]. For local communities living around protected areas, the significant drop in income from eco-tourism meant that in some areas, communities increased their reliance on natural resources (obtaining them illegally from the protected areas [20]) where conservations organizations did not have the means to increase their support. In other cases, donors shifted their giving to provide support to locally based, eco-tourism guide associations to try and counteract some of these impacts and also to ensure their continuity into the future (R. Mittermeier, pers. obs.).

The impact of the COVID-19 pandemic on other drivers of biodiversity loss is less clear. For example, in regard to the hunting of wild animals, some studies found that wildlife consumption declined in high demand countries (for example, China −28%, Thailand −41%, Vietnam −39%), with nearly half of people surveyed indicating that their decreased consumption was related to concerns about zoonotic disease transmission [21]. In other countries, however, there were reports of increased hunting and poaching including in Cambodia [22], Indonesia (M. Friis Hansen, unpubl. data), Madagascar [19], and Mexico [23]. Regarding the live trade of wildlife, studies are likewise mixed in their findings. One study found virtually no mention of COVID-19 in more than 20,000 online wildlife trade advertisements [24], although others hypothesized there could have been increased demand for live primates [25,26]. A systematic survey of online trade in two species of macaques saw a steep increase in macaques offered for sale on Facebook in Indonesia at the beginning of the pandemic, and this has continued ever since (M. Friis Hansen, unpubl. data). All this within a broader backdrop in which some parts of the world saw a large increase in pet ownership during COVID-19 [27] while, in others, a “pathological fear” developed of companion animals due to worries about disease transmission [28].

As a whole, the available evidence points to a picture in which biodiversity in many parts of the world has been and is still being negatively impacted by the COVID-19 pandemic, and where these negative impacts have become harder to manage. These negative impacts are often indirect and quite complex, and linked to human development issues such as food and water security, as well as governance and political systems [29]. Shifts in their magnitude depend, therefore, on the local and national context. It is not surprising that the United Nations has indicated that the achievement of the Sustainable Development Goals (SDGs) will be hampered by the impacts of COVID-19 [30], including SDG15 (Life on Land). The SDGs, also known as the Global Goals, were adopted by the UN General Assembly (which is comprised of the 193 member states of the United Nations) in 2015 to promote global sustainable economic development by 2030. The 17 goals expanded upon the 8 Millennium Development Goals and are novel in their cross-cutting and interdisciplinary nature. Though it will take all actors in society to work together to achieve the SDGs, primary responsibility sits with national government as the signatories of the agreement.

Here, we use non-human primates (hereafter referred to as ‘primates’) as a case study to examine the impacts of COVID-19 on society’s ability to achieve its sustainability targets in relation to biodiversity conservation and management under SDG15, with particular reference to SDG indicators 15.5.1, which uses the International Union for Conservation of Nature (IUCN) Red List of Threatened Species to assess risk of biodiversity extinction, and 15.1.2 and 15.4.1, which are related to the area of land under formal protection (Table 1). Primates provide an interesting case study not only because they are a particularly well-studied group of animals that are often the target of on-the-ground conservation initiatives (e.g., [31]), but also 63% of all primates are today classified as threatened with extinction on the IUCN Red List [32].

Perhaps most importantly, primates are not only indirectly susceptible to the impacts of the COVID-19 pandemic but also directly susceptible to the SARS-CoV-2 variant of coronavirus that causes COVID-19 [34]. Several lemur species are considered high risk [32], which is concerning as some species are unable to survive in captivity, and lemurs (Lemuriformes) are the most threatened of the larger groups of mammals—106 of the 112 species and subspecies (95%) are now categorized as threatened on the Red List [35]. The platyrrhine primates (those native to Central and South America) show decreased susceptibility to SARS-CoV-2 [34] and to in vivo pathology [36]. All catarrhine primates (African and Asian monkeys and apes) are susceptible to SARS-CoV-2 [34,37], and in vivo experiments in some monkeys have demonstrated their infection (*Macaca mulatta*, *M. fascicularis*, *Papio hamadryas*, *Chlorocebus sabaeus*) [34].

The susceptibility and severity of pathology varies by primate species [36]. When the pandemic was first declared, the IUCN Species Survival Commission (SSC) issued a statement recommending emergency measures be implemented at all great ape tourism and research sites [38] and similar initiatives were launched by regional organizations including in Brazil (e.g., [39,40]). In 2021, the first case of COVID-19 in a great ape was diagnosed in captive western gorillas (*Gorilla gorilla*) at San Diego Zoo Safari Park in 2021 [41]; subsequent outbreaks in gorillas in several other zoos have been confirmed. Given the impact of COVID-19 directly and indirectly on primates, it is important to understand how the pandemic has impacted our ability to protect and manage biodiversity for their benefit.

## 2. Materials and Methods

### 2.1. Online Survey

In January 2022, we sent an English-language online survey to members of the IUCN Species Survival Commission (SSC) Primate Specialist Group (PSG), a group of more than 700 experts across the world. Members of the PSG are considered authorities regarding primate conservation initiatives on-the-ground, including experts in both range and non-range countries. The voluntary, 20 min survey asked PSG members to give their opinion on the impact of the COVID-19 pandemic on their ability to do their primate-related work, as well as on protected areas in primate range countries, on primate hunting, and on live primate ownership. These topics were included in the survey because of their direct relevance to SDG15 indicators and targets (Table 1). PSG members were invited twice to complete the survey, with 93 having done so by mid-March 2022. The survey solicited this information through a series of closed and open-ended questions (File S1). Many questions used Likert Scale responses which are often used to measure attitudes and opinions [42].

### 2.2. Ethical Research Considerations

Research was deemed exempt by an ethics oversight committee (Institutional Review Board, University of San Diego, 2022). All survey participants were adults over the age of 18. Only current members of the Primate Specialist Group were recruited to participate in this survey.

### 2.3. Analysis

Results are presented as mean values with standard deviations. We examined the difference in responses between respondents living in primate range and non-range countries using Fisher’s Exact Tests. Due to the voluntary nature of the survey, sample sizes varied but are clearly noted where relevant.

## 3. Results

### 3.1. About the Survey Respondents

Ninety-three PSG experts responded to the survey. They collectively worked on 262 primate taxa (out of 713 taxa currently recognized by the Primate Specialist Group; 5 ± 5 taxa per respondent). These respondents were from 30 countries on all continents except Antarctica. Just over half (56%) of the surveyed respondents lived in primate range countries. Just under one-fifth of the respondents (18% of 93 respondents) were based in the United States of America.

Respondents were affiliated with a range of institutions: 51% with academia, 39% with non-profit organizations (local and international) and social enterprises, 7% with governments, 5% with zoos, and 2% with field stations. Almost one out of ten (9%) surveyed experts had changed their institutional affiliation due to the COVID-19 pandemic.

### 3.2. Impact of the COVID-19 Pandemic on the Ability of Primate Experts to Work on Primate-Related Initiatives

Nine out of ten (90%) respondents had to work remotely from home at any point between March 2020 and March 2022, and this did not differ between respondents in primate range and non-range countries (85% of 52 respondents in range countries vs. 98% of 41 respondents in non-range countries; Odds Ratio = 0.14; Fisher’s Exact Test, *p* = 0.0724, *n* = 93). Two-thirds of respondents (67%) said the institution they were affiliated with had closed partially or completely in that same time period due to the COVID-19 pandemic, with respondents in primate range countries 1.58 times more likely to report a partial or complete closure of their workplace (71% of 52 respondents) than respondents in non-range countries (61% of 41 respondents, Fisher’s Exact Ratio, *p* = 0.3768, *n* = 93 respondents). Respondents explained that they had to stop field activities several times because of confinement orders, though in two cases, it was not a respondent’s institution that closed, but the protected area that he/she was working in. These closures resulted in lost income for institutions. For example, one respondent wrote: “Our [non-governmental organization (NGO)] runs a program where international students pay bench fees to conduct their own research projects at our sites under the supervision of our professional scientists. This program was completely suspended for eight months due to border closures”. COVID-19 pandemic restrictions continue to impact respondents; 58% were working remotely or from home due to the COVID-19 pandemic at the time of taking the survey, and 26% said their institution was partially or completely closed at the time of taking the survey.

Four out of ten respondents (39%) had not been able to visit their field sites since March 2020 due to the COVID-19 lockdown, and a further one out of ten (10%) had only been able to visit some of their field sites in that time. Respondents in non-range countries were 5.43 times more likely not to have been able to visit all of their field sites since March 2020 than respondents in range countries (29% of 41 non-range country respondents vs. 69% of 52 range country respondents, Fisher’s Exact Test, *p* = 0.0002).

Four out of five (80%) respondents said that they, or the institution they were affiliated with, could put adaptive measures in place to mitigate or minimize the impact of the COVID-19 pandemic on their primate-related work. Respondents in range countries were 2.67 times as likely to say that adaptive measures were possible (87% of 52 respondents) compared to respondents in non-range countries (71% of 41 respondents, Fisher’s Exact Test, *p* = 0.0734). Respondents noted different actions that had been/could be taken including: being flexible about when they undertake field work, engaging local conservationists, and a range of standard COVID-19 reduction measures (e.g., home working, use of face coverings, support staff and their families to get vaccinated).

Since March 2020, 54% of respondents said the amount of funding they had available for their primate-related work was now lower or much lower, and this appeared to affect both range and non-range respondents equally (56% and 51% of range and non-range respondents, respectively; Fisher’s Exact Test, *p* = 0.6810). Only 36% said they had the same amount of funding available to them, and 9% said they had higher or much higher amounts of funding available. It was not just amounts of funding that had changed, but also the reliability of that funding to flow; half (50%) of respondents said the amount of funding for their primate-related work had been stopped or interrupted by the COVID-19 pandemic, with 50% of both range and non-range respondents having experienced such changes in funding reliability (Fisher’s Exact Test, *p* = 1.00). In some cases, the change in funding streams was indirectly due to COVID-19. For example, two respondents said they applied for less funding because of the pandemic. In another case, one respondent noted that sources of private sector funding (e.g., Corporate Social Responsibility funding) had been diverted away from their work and towards COVID-19 efforts.

**Figure 1:**
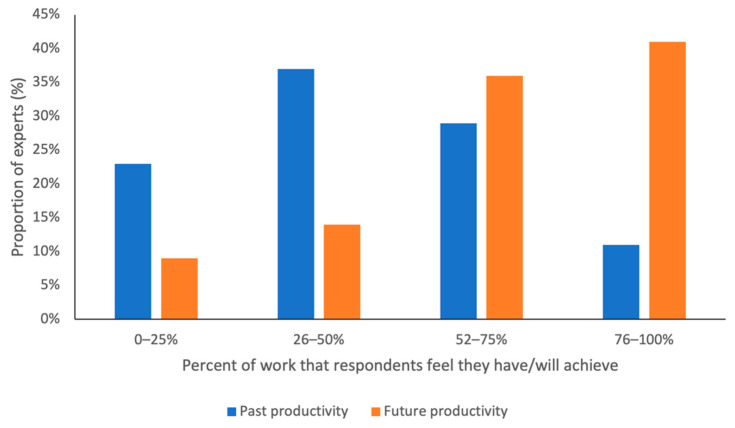
Self-reported work productivity by respondents in the past (from March 2020 to March 2022) and into the future (into the next two years). Respondents were asked to estimate how much they had managed to achieve (from four categories) relative to what they would have achieved had the COVID-19 pandemic not occurred.

Respondents surveyed had not been able to achieve as much of their primate-related work as they had hoped, since the onset of the COVID-19 pandemic. Only one out of ten respondents (11%) said they had managed to achieve 76–100% of the primate-related work since March 2020, that they had planned prior to the COVID-19 outbreak (Figure 1). Looking forward, four out of ten respondents (41%) expect to be able to complete 76–100% of the primate-related work they previously planned.

All but two respondents (91 out of 93 people) responded when asked to describe how the COVID-19 pandemic had affected their ability to conduct primate-related work. Many described the difficulties mentioned above (remote working, reduced funding, difficulty travelling to field sites, delays in progressing their work), with one respondent describing being in 400 days of lockdown and several describing their country/field sites as being virtually inaccessible for well over 1.5 years. In other cases, the closure of national parks meant that primatologists could go about their everyday lives, but not undertake their primate-related work. One respondent simply wrote, “No research. No tourism. [It’s] devastating. For two years no students [and] no researchers worked at our research station”. Aside from the physical and emotional impact of COVID-19 on themselves and their staff, they described:

1. Additional administrative workloads from the pandemic taking away from their ability to do substantive primate-related work (three respondents) and working on a reduced salary (two respondents);
2. Complete/permanent closures of programs (two respondents) or pausing programs to safeguard local communities (two respondents) or primates (two respondents) against increased disease risk;
3. Increased financial costs due to COVID-19 testing and purchase of personal protective equipment (PPE) (two respondents) and increased time needed to quarantine prior to entry to field sites (two respondents);
4. Research permits expiring and taking unusually long periods of time to be renewed due to the impacts of COVID-19 on governments (one respondent) or inability to export/import samples for months/years following the onset of the pandemic (one respondent);
5. Two respondents said that the urgency for COVID-19 vaccinations or test processing has impacted on their work (e.g., laboratories being re-purposed away from offering a range of analytical services to focusing on COVID-related analyses);
6. Breakdown of technical equipment in the field that could not be repaired due to lack of accessibility as a result of COVID-19 travel restrictions (one respondent);
7. Long-term data collection disrupted, with one respondent writing, “we have 35 years of continuous primate follows but in 2020 we only have a few months of data”;
8. Primates becoming unhabituated to respondents (one respondent);
9. The risk of following habituated primates in the wild being too high due to disease transmission (one respondent);
10. Delays for both range-country and non-range country students in obtaining their university degrees due to lack of ability to do field research (two respondents) and student field courses being cancelled (four respondents);
11. Fewer discussions and exchanges with local leaders adjacent to/near project areas (one respondent).

There were a few positive changes that respondents mentioned. For example:

1. When the pandemic started in March 2020, one respondent’s students were safer staying at the field site than returning to crowded cities;
2. Two respondents were able to expand their consulting services due to the wider acceptance of digital working;
3. Four respondents described their in-country colleagues and staff as taking on a greater leadership role in projects, or local communities strengthening their participation in projects;
4. One respondent described their organization proactively using the time to rebuild ageing tourism infrastructure;
5. Several noted that they were able to publish more articles than usual, working with their existing datasets.

### 3.3. Impact of the COVID-19 Pandemic on Protected Areas

**Figure 2:**
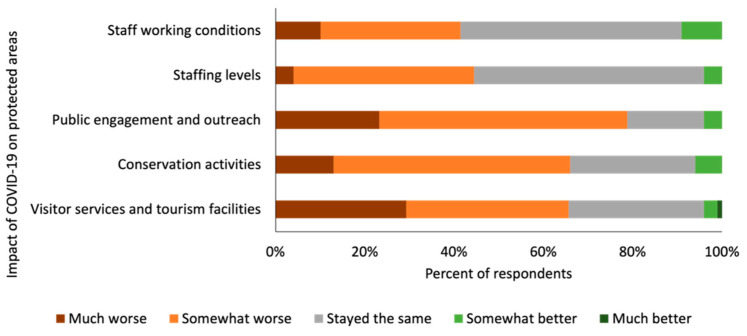
Opinions given by respondents on the how the COVID-19 pandemic had affected different aspects of protected area functioning and governance.

Of the respondents surveyed, 80% (*n* = 75 out of 93 respondents) did primate-related work that involved working in/around protected areas. These respondents generally felt that the services provided by protected areas were worse than before the COVID-19 pandemic (Figure 2). Six out of ten respondents (61% of 70 respondents) felt that primate conservation in the protected area(s) where they worked was ‘somewhat worse’ or ‘much worse’. In relation to the protected areas where respondents did primate-related work:

1. Two-thirds (65% of 69 respondents) felt that visitor services or tourism facilities at protected areas were ‘somewhat worse’ or ‘much worse’;
2. Two-thirds (66% of 68 respondents) felt that conservation activities, such as patrolling, anti-poaching, monitoring, research, control of invasive species, and habitat restoration are ‘somewhat worse’ or ‘much worse’;
3. Eight out of ten (78% of 69 respondents) felt that public engagement, outreach and the provision of services to local communities in and around the protected areas were ‘somewhat worse’ or ‘much worse’;
4. Four out of ten (44% of 67 respondents) felt that protected area staffing levels were ‘somewhat worse’ or ‘much worse’;
5. Four out of ten (42% of 67 respondents) felt that working conditions, workloads, safety or well-being of protected area staff were ‘somewhat worse’ or ‘much worse’;
6. Over half (61% of 67 respondents) felt that the financing of protected areas was ‘somewhat worse’ or ‘much worse’.

Respondents were asked what measures were introduced in protected areas in response to the COVID-19 pandemic, that will be continued after the pandemic is over. Half of the respondents (52% of 63 respondents) said they were not sure, or that there were no measures in place after the pandemic. The other half, however, listed a range of protective measures including: (1) new or improved health protocols to reduce disease transmission from humans to primates (27% of respondents) including the use of face masks, restricted visitor numbers, and minimum distancing with primates; and (2) more or different types of patrolling (10%) including the increased use of local communities in patrolling. Individual respondents also said that they thought there would be an increase in the use of technology to do remote protected area monitoring and an increase in other remote work.

Respondents were asked what lessons for protected areas can be learned from the COVID-19 pandemic, and how protected area management should be changed in the post-COVID-19 era. One-quarter (28% of 57 respondents) mentioned a need to adjust protected area funding models and one-quarter (25% of 57 respondents) mentioned the need to improve governance and operations of protected areas. Several respondents mentioned, for example, the need to diversify funding sources (across state and non-state actors), and described the need to move away from a reliance on funding from tourism. One respondent even wrote, “we did not rely on tourism before [the pandemic] and I think that has been essential in being able to continue our project”. In regard to governance and operations, respondents noted the importance of having consolidated systems, decentralized staffing (e.g., use of staff in proximity to the protected area; establishment of local community groups to continue monitoring and management), improved protocols (for management, patrol, risk management, and monitoring), long-term/multi-year and sustainable financial and governance plans, improved facilities (technology, infrastructure, and programming), and adaptive management.

Improved tourism management (through, for example, reduced numbers or introduction of virtual tourism) was mentioned by four respondents (7%), while five respondents (9%) mentioned the need to think about alternative livelihoods for local communities or consider how local communities were engaging with the protected area(s). Six respondents (11%) mentioned the need to continue to implement health protocols that protect primates.

### 3.4. Impact of the COVID-19 Pandemic on Primate Hunting

**Figure 3:**
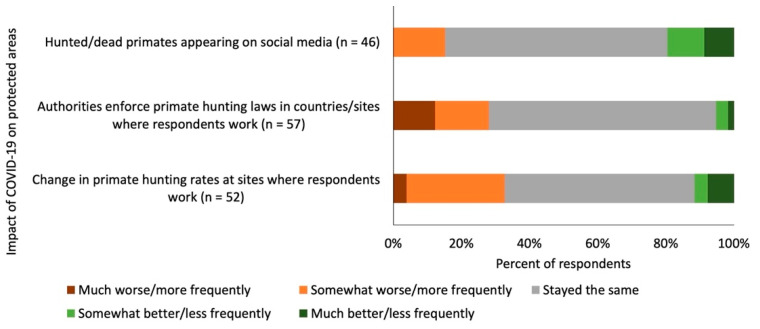
Opinions given by respondents on the how the COVID-19 pandemic had affected different aspects of primate hunting in sites and countries where they worked.

Four out of ten respondents (39% of 93 respondents) did not know whether primate hunting had changed since the onset of the COVID-19 pandemic in March 2020. This included more than half of respondents (51% of 41 respondents) in non-range countries but less than one-third of respondents (29% of 52 respondents) in non-range countries (odds ratio: 0.39; Fisher’s Exact Test, *p* = 0.0336). Of the 52 respondents who had a view on whether or not hunting practices had changed following the onset of COVID-19, 56% felt that hunting rates had not changed, 33% felt hunting was happening ‘more frequently’ or ‘much more frequently’, and 12% felt that hunting was happening ‘less frequently’ or ‘much less frequently’ (Figure 3).

One-third of respondents (32% of 87 respondents) did not know whether authorities had changed how effectively they enforced laws in regard to primate hunting in the sites/countries where they conducted their primate-related work. Of the 59 respondents who did have a view on the situation, two-thirds (64%) felt that law enforcement effort was the same as before, with only 5% saying it was ‘somewhat better’ or ‘much better’ and 27% saying it was ‘somewhat worse’ or ‘much worse’ (Figure 3). In regard to the coverage of hunting on social media, half of respondents did not know (47% of 87 respondents) whether or not hunted/dead primates were appearing more or less frequently on social media since the onset of the COVID-19 pandemic in March 2020. Of the 46 respondents that had a view, two-thirds (65%) said the situation was the same as before, 20% said it was happening ‘less frequently’ or ‘much less frequently’, and 15% said it was happening ‘more frequently’ (Figure 3).

Respondents were asked why they believed COVID-19 had changed or not changed the hunting of primates at the sites where they work. In their view, hunting had increased because of lower levels of oversight including fewer patrols, less active research, and fewer tourists visiting (13 respondents). One respondent wrote bluntly, “[Our] personnel followed COVID restrictions. Poachers did not”. Hunting was also thought to have increased because of food security issues (including increase in food prices) and due to lack of alternative income often because of a lack of tourism or because of disruption in food commodity trade networks (11 respondents). In India and Cambodia, hunting was noted to have increased at the start of the pandemic when people temporarily moved from urban areas back to rural areas. In cases where respondents saw no change in hunting rates, they said it was either because hunting simply continued as normal (5 respondents), or because there were extenuating circumstances as to why hunting was not common in the first place including, for example, religion (1 respondent), the small size of the primates (1 respondent), and the presence of criminal groups operating in the region (1 respondent).

Respondents were asked if they knew of any primates that had been killed specifically due to COVID-19, for example due to fear that primates were carriers of COVID-19. No respondents reported having heard about any primate deaths directly due to COVID, though in one case, a respondent wrote, “we noticed that there were *A[louatta] pigra* monkeys with coughs and sneezes at the same time as the peaks of contagion in the communities…[but] the death of primates has not increased”.

### 3.5. Impact of the COVID-19 Pandemic on Live Primate Ownership

**Figure 4:**
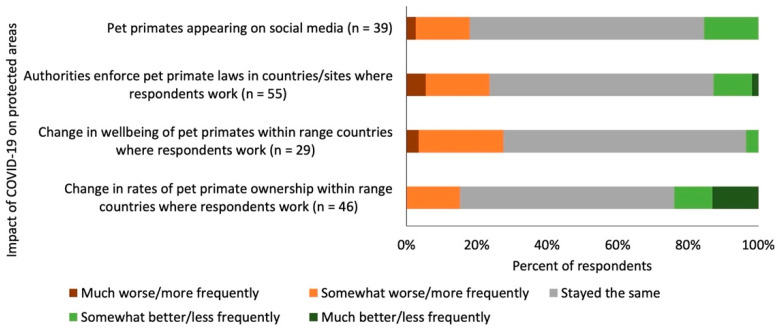
Opinions given by respondents on the how the COVID-19 pandemic had affected different aspects of pet primate ownership in sites and countries where they worked.

Most respondents did not know whether the COVID-19 pandemic had changed the frequency with which primates were being kept as pets (49% of 90 respondents). Of the 46 respondents with a view, 61% thought the situation was the same as before, 24% thought it was happening ‘less frequently’ or ‘much less frequently’, and 15% thought it was happening ‘more frequently’ (Figure 4). One respondent wrote that in Indonesia, “during the early months of the pandemic, the interest of keeping pets/wild animals increased, creating [an] additional market for wildlife and encouragement for poaching”. In another case, a respondent provided anecdotal information that chimpanzee (*Pan troglodytes*) orphans had been confiscated in greater numbers since the pandemic started. The majority of respondents did not know if the COVID-19 pandemic had changed the wellbeing of primates kept as pets within habitat range countries (67% of 89 respondents; though 69% of the 29 respondents who had a view said that pet primate wellbeing ‘stayed the same’).

Four out of ten respondents did not know whether authorities had changed their effectiveness in enforcing the laws with regard to pet primate ownership in the sites/countries where they conducted their primate-related work (38% of 89 respondents). Of the 55 respondents that had a view, 64% said the situation was the same as before (though several commented that enforcement had already been so poor before the pandemic, so perhaps it could not get worse than it already was), 24% said it was ‘somewhat worse’ or ‘much worse’, and 13% said it was ‘somewhat better’ or ‘much better’ (Figure 4).

In regard to the coverage of pet primates on social media, respondents often did not know (55% of 89 respondents) whether or not pet primates were appearing more or less frequently on social media since the onset of the COVID-19 pandemic in March 2020. Of the 40 respondents that had a view, 65% said the situation was the same as before, 18% said it was happening ‘more frequently’ or ‘much more frequently’, and 15% said it was ‘less frequently’ (Figure 4).

One out of ten respondents were aware of a pet primate being released into the wild, sold, killed, or given away as a gift due to COVID-19 (10% of 89 respondents were aware of such an incident).

## 4. Discussion

### 4.1. Impact of the COVID-19 Pandemic on Primate Experts’ Ability to Work on Primate-Related Initiatives

As with many other industries and professions, the COVID-19 pandemic impacted the respondents’ ability to progress in their primate-related work. We found that respondents in both primate range and non-range countries experienced professional difficulties due to COVID-19, and both reported, for example, similar difficulties accessing funding. Both sets of respondents also reported drastically reduced productivity as COVID-19 interrupted travel and research agendas. For example, to prevent primates from contracting COVID-19 from humans, many primate-viewing destinations (national parks and other protected areas) were temporarily closed to visitors (e.g., some parks in Gabon, Nigeria, and the Republic of Congo; parks managed by Madagascar National Parks; all protected areas in Indonesia) and researchers. In some cases, respondents reported voluntarily pausing their work so as not to potentially expose primates to COVID-19. If our survey results—which found that only one out of ten respondents had achieved most of their planned primate-related work since March 2020—are representative of wider progress on biodiversity research and conservation initiatives, it does not bode well for wider progress under SDG15. The slowdown of research and conservation initiatives, coupled with an inability to conduct fieldwork, has surely had an economic impact on primate habitat countries, many of which are heavily dependent on tourism (including from researchers) for revenue.

It is interesting, though not surprising, that respondents in non-range countries experienced different types of difficulties than those in range countries and, perhaps consequently, the information they could provide differed. For example, respondents in range countries were more than five times more likely than respondents in non-range countries to have visited all of their field sites since March 2020, and almost three times more likely to say that adaptive measures were possible to ensure their primate-related work continued. Likewise, respondents in non-range countries were twice as likely to say, for example, that they did not know whether primate hunting in their study sites had changed since the onset of the COVID-19 pandemic. One leading primate respondent based in Europe whose career exceeds 45 years—and after having selected the response “I do not know” in almost every question of the survey—closed out their survey response simply by writing, “it is amazing how little we actually know”. Here, as elsewhere in this paper, it is important to acknowledge that the anecdotal observations captured within our survey may or may not reflect the overall trends in threats facing primates (e.g., trends in primate hunting or pet ownership) though many of the observations reported in the survey are concerning (see below).

It is important to note that these differences between range and non-range primate respondents will impact conservation efforts differently in different parts of the world. For example, Neotropical primates tend to be studied proportionately more by range-country primatologists than African primates are, and hence Neotropical primate research and conservation efforts were perhaps more able to adjust to the COVID-19 pandemic than other geographies where most primatologists are from non-range countries. This may also be why our survey was responded to by respondents from just one African country (Madagascar), as compared to the other geographies, where we had responses from seven Asian countries and ten South American countries. A lesson learned here is to ensure the sustainability of research sites long term, including having exit plans in place [43], and to more proactively address broader social and ethical issues that arise in the course of tropical research and conservation agendas [44]. These exit plans should protect the local communities and primate population if events such as the COVID-19 pandemic occur. If it is not possible to have exit plans or to commit long-term to a site, researchers may need to reconsider initiating research [45].

A positive outcome, as described by respondents, is the increased collaboration between range and non-range country respondents, and with local communities. This increased collaboration and increased inclusion of range country respondents and local communities is a very positive trend and bodes well for the future of primate conservation and research, representing a possible permanent shift in the primatological community which has been noted by others [46,47].

### 4.2. Impact of the COVID-19 Pandemic on Protected Areas

The importance of effective protected areas to the delivery of SDG15 is evidenced in their inclusion in SDG15 indicators (15.1.2; 15.4.1; Table 1). It is important, therefore, that six out of ten respondents in our study felt that primate conservation in protected areas was worse than before the onset of the COVID-19 pandemic. When asked about six different aspects of protected area governance and management, more than half of respondents felt that the protected areas were worse off in four out of the six areas (including visitor services, patrolling and anti-poaching activities, provision of services to local communities, and in terms of the financing of the protected area).

These results are concerning, not least because primates are charismatic megafauna that can generate significant resources for conservation and serve as flagship species for governmental and stakeholder aspirations and initiatives (e.g., [48]). In other cases, some primates are now entirely restricted to protected areas (e.g., mountain gorilla subspecies *Gorilla beringei beringei*) and protecting the integrity of these habitats is crucial. If the information collected in our survey reflects reality ‘on-the-ground’, it will take much more resource and significant effort to recover from the damage incurred over the last two years. Funding constraints were unfortunately described by numerous respondents, many of them proposing contradictory solutions: where government funding was lacking, they proposed that this needed to be secured, and where government funding was the sole funding source, they proposed that funding streams needed to be diversified. All this in the context that, even before COVID-19, protected areas in less-developed countries were experiencing higher anthropogenic pressure [49]—often because local communities’ livelihoods were based on subsistence living practices—and so were already in need of additional resources and support. This points to a need for primate conservation projects, both within and outside protected areas, to move towards diversified portfolios of funding to buoy these initiatives against the deleterious effects of sudden drops in tourism-related income.

Helpfully, of the respondents in our survey who had an opinion on how protected area management could be improved, there was a clear consensus on the importance of continuing and strengthening health protocols and of diversifying and improving patrols and monitoring. This is important as it relates not just to the COVID-19 pandemic but to other communicable diseases such as the Avian influenza. Prior to the COVID-19 pandemic, disease prevention measures had been elaborated for primate tourism and research (e.g., [50,51]). Still, appreciation for the importance of these measures was low even in the primatology community. Prior to the pandemic, for example, disease prevention measures were not routinely promoted at lemur-watching sites in Madagascar despite evidence of human-lemur disease transmission (e.g., [52,53]). This meant that popular lemur-watching sites were over-crowded and minimum distancing was not observed, with lemurs in some sites continually disturbed by human visitors (J. Ratzimbazafy, pers. obs.). There is an opportunity now to ‘reset’ primate tourism in Madagascar and address these issues, so as to make it more sustainable. Likewise in Brazil, it was only after COVID-19 that research permits included precautionary recommendations for researchers to limit disease transmission. In Central Africa, the wearing of face masks by great ape tourists in Virunga National Park (Democratic Republic of the Congo) has been required for more than a decade, but adoption of the IUCN best practice guidelines has now improved at many sites. Post-COVID, additional measures have been put in place (handwashing stations constructed at tourist reception points, skin temperature of tourists measured, proof of vaccination and/or negative COVID test result required), and the wearing of face masks by great ape tourists and researchers has become obligatory in Rwanda and Uganda.

Respondents also provided a good range of tangible governance and management improvements to institute, with many emphasizing the need to develop interdisciplinary programs to support local communities. Research has also shown that community-managed forests experience less deforestation than protected ones [54] further indicating a need for a shift in primate conservation.

### 4.3. Impact of the COVID-19 Pandemic on Primate Hunting

Our survey showed that—where respondents felt that COVID-19 had changed hunting rates—they were more than two times more likely to say that hunting had increased rather than decreased due to COVID-19. While the anecdotal observations of experts in our study may not reflect overall changes in hunting patterns, the diversity in responses reflects, however, that wildlife trade markets and wildlife commodity chains are structured very differently in different countries (e.g., see [55] in Madagascar), and also that primate meat is eaten not just for food security reasons but also as a result of cultural preference. This meant that we sometimes received seemingly contradictory information from respondents. For example, in one case, the closure of food markets increased food insecurity which increased primate hunting (as people hunted primates in order to feed themselves). In another case, however, the closure of food markets and transit routes reduced primate hunting because the commodity chain had been disrupted and this then reduced demand from middlemen in the wild meat trade. One-quarter of respondents felt that the authorities were enforcing hunting laws less than before the pandemic.

Extraction of primates from the wild can include hunting of primates for meat or extraction of live animals for pet ownership, entertainment, and research. While it is still difficult to conclude how the ongoing pandemic has changed the extraction of primates from the wild, reports from Southeast Asia and Colombia confirm an increase in hunting of primates, especially macaques, for research, both nationally and internationally (M. F. Hansen and A. Maldonado, pers. obs.). In Bangladesh, a local pharmaceutical company turned to wild rhesus macaques (*Macaca mulatta*) for preclinical COVID-19 vaccine testing, which lead to a local outcry. The demand for non-human primates for preclinical testing has undoubtedly increased during the COVID-19 pandemic, and further threatens wild primate populations [56].

### 4.4. Impact of the COVID-19 Pandemic on Primate Pet Ownership

Our survey showed that—where respondents felt that COVID-19 had changed primate pet ownership rates—they were more likely to say that pet ownership had decreased than increased. It was also clear that on this topic, respondents were far more likely not to know the answers to our questions, than when asked similar questions about primate hunting or protected area governance. Prior to the COVID-19 pandemic, it was already the case that in some regions, the magnitude and scope of hunting and capture of primates was not accurately reflected in the peer-reviewed literature because of the difficulty in researching oft-illegal extraction and trade (e.g., African lorises, [57]; lemurs, [58]). In some countries, the trade and ownership of pet primates is so hidden that not even neighbors of primate owners are aware that there is a pet primate in their vicinity (e.g., lemurs; [59]). In the context of COVID-19, which further restricted people’s movements within their national borders, it is perhaps not surprising that knowledge of this subject area is low among the respondents to our survey.

Given the extensive closures of international borders, our assumption had been that during the first two years of the pandemic, any increases in the trade of live pet/captive primates would have been seen domestically (i.e., increases contained within primate range countries). Counterintuitively, however, the most significant example of increased live extraction of primates was reported for the “the use of wild primates (capture and transportation across international borders) to test medicines by labs against COVID” and was described as, “increasing and…being excused by the argument that new medicines need to be developed to fight COVID”. This anecdote is supported by data which show that from 2019 to 2020, international primate trade of the long-tailed macaque (*Macaca fascicularis*) increased 225% from 61,000 individuals traded to 151,000 individuals [60] due to the demand for primate research subjects for COVID-19 pre-clinical research and toxicology testing [25,26]. Many of these primates are suspected to have been wild-caught. Concurrently, both price and demand for *M. fascicularis* as a trade commodity have skyrocketed during the COVID-19 pandemic relative to the already regular and heavy pre-pandemic capture and trade [25,26]. The price per long-tailed macaque quadrupled from 2019 [26] to 2022. Should we see this trend in other primate species, the trade of live animals could represent a significant new threat and would directly impact on countries’ abilities to make progress towards SDG 15.5.1 (Table 1).

## 5. Conclusions

This study provides further evidence that the impacts of COVID-19 have likely jeopardized progress towards SDG15. First, our study suggests that primates are likely to be facing increased threats due to the impacts of COVID-19. Respondents in our study felt that protected areas with primate populations were broadly doing worse following the onset of the pandemic, and many reported increases in primate hunting and the primate pet trade. In other cases, respondents to our survey listed a range of ways in which primates been impacted indirectly by COVID-19, including: (1) through habitat loss following increased agricultural production to address COVID-19-related food security issues; (2) where COVID-19 has been used as a pretext to weaken environmental protection (e.g., in Brazil); or (3) where COVID-19 simply distracted funders, governments, and other stakeholders away from environmental topics and towards health issues. All of these pieces of evidence, combined with what we already knew to be a difficult primate conservation and management landscape, do not paint a positive picture with regard to the SDG15.5′s aim to protect and prevent the extinction of species (Table 1).

Addressing the impacts of COVID-19 on primate conservation and management initiatives will require more funding, although this begs the question from where this additional funding will come. Zoos, which fund a lot of primate work around the world, have seen their budgets drastically reduced due to lack of visitors during COVID-related closures. One respondent wrote that their zoo, “closed for a total of 242 days in 2020/2021 [and this] led to reduced funding of our primate projects. The same happened to zoos globally, so that primarily zoo-funded projects suffered considerably financially”. For zoos, it is not just the number of days they are open for visitors to consider, but also how “extended periods of visitor absence and changes in human behavior have affected and potentially continue to affect animal behavior”. There are similarly complex questions surrounding funding from other sources such as governments, the private sector, and high-net-worth individuals and private foundations. Although colleagues may have partially offset budget reductions through cost savings achieved by remote participation in workshops and conferences, it is not clear that this is good long-term budget management strategy.

There are opportunities, however, to help engage the public in primate conservation. In many areas of the world, the COVID-19 pandemic helped reconnect people to nature and their natural surroundings, or represents an opportunity to enhance biodiversity conservation [14,16]. In one touching example, a respondent shared how an NGO in a primate range country had created a small remembrance forest where, “friends and relatives can plant native-tree seedlings to honor the memory of loved ones lost to COVID-19, moving many to tears”. The person concluded by noting that, “people who planted native trees there will value that forest forever”.

Lastly, the COVID-19 crisis has been an opportunity to reassess the management and research strategy approaches for biodiversity conservation, particularly in low-income regions [16]. An inclusive approach is especially important when we consider that the wider primate conservation landscape typically goes beyond protected areas, and into areas where humans and primates must necessarily coexist.

## Figures and Tables

**Table 1:** Targets and Indicators under Sustainable Development Goal 15 (Life on Land) [33]. Indicators of particular relevance to this article are presented in bold font.

Goal 15. Protect, Restore and Promote Sustainable Use of Terrestrial Ecosystems, Sustainably Manage Forests, Combat Desertification, and Halt and Reverse Land Degradation and Halt Biodiversity Loss		
Target	Description	Indicator
15.1	By 2020, ensure the conservation, restoration and sustainable use of terrestrial and in-land freshwater ecosystems and their services, in particular forests, wetlands, mountains and drylands, in line with obligations under international agreements.	15.1.1 Forest area as a proportion of total land area. **15.1.2 Proportion of important sites for terrestrial and freshwater biodiversity that are covered by protected areas, by ecosystem type.**
15.2	By 2020, promote the implementation of sustainable management of all types of forests, halt deforestation, restore degraded forests and substantially increase afforestation and reforestation globally.	15.2.1 Progress towards sustainable forest management.
15.3	15.3 By 2030, combat desertification, restore degraded land and soil, including land affected by desertification, drought and floods, and strive to achieve a land degradation neutral world.	15.3.1 Proportion of land that is degraded over total land area.
15.4	15.4 By 2030, ensure the conservation of mountain ecosystems, including their biodiversity, in order to enhance their capacity to provide benefits that are essential for sustainable development.	**15.4.1 Coverage by protected areas of important sites for mountain biodiversity.** 15.4.2 Mountain Green Cover Index.
15.5	15.5 Take urgent and significant action to reduce the degradation of natural habitats, halt the loss of biodiversity and, by 2020, protect and prevent the extinction of threatened species.	**15.5.1 Red List Index.**
15.6	15.6 Promote fair and equitable sharing of the benefits arising from the utilization of genetic resources and promote appropriate access to such resources, as internationally agreed.	15.6.1 Number of countries that have adopted legislative, administrative and policy frameworks to ensure fair and equitable sharing of benefits.
15.7	15.7 Take urgent action to end poaching and trafficking of protected species of flora and fauna and address both demand and supply of illegal wildlife products.	15.7.1 Proportion of traded wildlife that was poached or illicitly trafficked.
15.8	By 2020, introduce measures to prevent the introduction and significantly reduce the impact of invasive alien species on land and water ecosystems and control or eradicate the priority species.	15.8.1 Proportion of countries adopting relevant national legislation and adequately resourcing the prevention or control of invasive alien species.
15.9	15.9 By 2020, integrate ecosystem and biodiversity values into national and local planning, development processes, poverty reduction strategies and accounts.	15.9.1 (a) Number of countries that have established national targets in accordance with or similar to Aichi Biodiversity Target 2 of the Strategic Plan for Biodiversity 2011–2020 in their national biodiversity strategy and action plans and the progress reported towards these targets; and (b) integration of biodiversity into national accounting and reporting systems, defined as implementation of the System of Environmental-Economic Accounting.
15a	15.a Mobilize and significantly increase financial resources from all sources to conserve and sustainably use biodiversity and ecosystems.	15.a.1 (a) Official development assistance on conservation and sustainable use of biodiversity; and (b) revenue generated and finance mobilized from biodiversity-relevant economic instruments.
15b	15.b Mobilize significant resources from all sources and at all levels to finance sustainable forest management and provide adequate incentives to developing countries to advance such management, including for conservation and reforestation.	15.b.1 (a) Official development assistance on conservation and sustainable use of biodiversity; and (b) revenue generated and finance mobilized from biodiversity-relevant economic instruments.
15c	Enhance global support for efforts to combat poaching and trafficking of protected species, including by increasing the capacity of local communities to pursue sustainable livelihood opportunities.	15.c.1 Proportion of traded wildlife that was poached or illicitly trafficked.

## Data Availability

The disaggregated dataset used in this study is not publicly available due to the duty of confidentiality to survey participants, and given that their responses could make them publicly identifiable. Survey participants, as part of their informed consent statement, consented to the following confidentiality: “The results of this research project may be made public and information quoted in professional journals and meetings, but information from this study will only be reported as a group, and not individually”.

## Supplementary Materials

The following supporting information can be downloaded at: https://www.mdpi.com/article/10.3390/ani12091214/s1, File S1. Survey questions used to collect data for this study.
