# Supplementary material for: Impact of the COVID-19 Pandemic on Primate Research and Conservation
Source: Animals (Basel). 2022 May 8;12(9):1214. doi: 10.3390/ani12091214 (PMC9099823; doi:10.3390/ani12091214)
Supplement: Supplementary file 1 [file animals-12-01214-s001.zip › animals-1669314-supplementary.pdf]

Supplementary Materials

Survey: Impact of COVID-19 on primate research and conservation

Online survey questions:

[preceded by the informed consent statement]

This survey will take 20 minutes to complete, depending on the depth and length of your responses. None of the questions are required, and you can stop participating in the survey at any time. Here, we ask you to think about answers in relation to your "primate-related work" which is broadly defined as paid or unpaid time (occasionally, part-time, or full-time) spent on research, volunteering, or conservation programming that involves primates.

Thank you very much in advance for making the time to respond to this questionnaire and for your contributions.

1. This survey is designed to understand the impact of the COVID-19 pandemic on primate research and conservation efforts across the world. Do you currently spend some of your time – either paid or unpaid – on research, volunteering, or conservation programming that involves primates (hereafter referred to as "primate-related work")? Please select 'yes' if you are a member of the Primate Specialist Group.
    a. *Yes [takes respondents to the next question of the survey]*
    b. *No [takes them to the end of the survey]*
2. Your name
    a. *Open response*
3. If you are affiliated with an organization or an institution, please provide the name of the organization/institution, as well as the role you have at this organization. This can include being a student, employee, or volunteer.
    a. *Open response*
4. Have you had to change the organization or institution that you are affiliated with, because of the COVID-19 pandemic?
    a. *Yes*
    b. *No*
    c. *Other*
5. Briefly describe the primate-related work that you or your organization undertakes.
    a. *Open response*
6. At the time of taking this survey, in what country do you permanently reside?
    a. *Drop-down menu of countries*
7. Please list the primate habitats/areas where your primate-related work takes place. Please provide the names of any landscapes or protected areas that you are working in (or worked in before COVID-19), and please list all countries where you work with primates.
    a. *Open response*
8. Please select the primate species that you work with.

    a. *Select all that apply [from a drop-down list of recognized primate taxa]*

Impact of COVID-19 on your ability to work on primate-related initiatives:

1. Have you had to work remotely or work from home at any point in time since March 2020 due to the COVID-19 pandemic?
    a. *Yes*
    b. *No*
    c. *Other*
2. At the time of taking this survey, are you working remotely or working from home due to the COVID-19 pandemic?
    a. *Yes*
    b. *No*
    c. *Other*
3. Did the organization or institution that you are affiliated with (in relation to your primate-related work) partially or completely close at any point in time since March 2020 due to the COVID-19 pandemic?
    a. *Yes*
    b. *No*
    c. *Other*
4. At the time of taking this survey, has the organization or institution that you are affiliated with (in relation to your primate-related work) partially or completely closed?
    a. *Yes*
    b. *No*
    c. *Other*
5. In relation to your primate-related work, have you been able to visit your field and project sites since March 2020?
    a. *Yes*
    b. *No*
    c. *Other*
6. How has the COVID-19 pandemic affected your ability to conduct your primate-related work? Feel free to include direct and indirect impacts.
    a. *Open response*
7. Can you, or have you or the organization/institution you are affiliated with, put adaptive measures in place to mitigate or minimize the impact of the COVID-19 pandemic on your primate-related work?
    a. *Yes*
    b. *No*
    c. *I don't know*
    d. *Other*
8. Since March 2020 (the onset of the COVID-19 pandemic), the amount of funding for your primate-related work is now:
    a. *Much higher*
    b. *Higher*
    c. *About the same*
    d. *Lower*

    e. *Much lower*

9. Since March 2020, has the flow of funding for your primate-related work been stopped or interrupted because of the COVID-19 pandemic?
    a. *Yes*
    b. *No*
    c. *Other*
10. Since March 2020, what % of the primate-related work, that you had planned before COVID-19, have you managed to complete as planned?
    a. *0-25%*
    b. *26%-50%*
    c. *51%-75%*
    d. *76%-100%*
11. In 2022, what % of the primate-related work (as compared to what you would have done before COVID-19) do you expect to continue?
    a. *0-25%*
    b. *26%-50%*
    c. *51%-75%*
    d. *76%-100%*

Impact of COVID-19 on protected areas:

12. Does your primate-related work involve working in/around protected areas?
    a. *Yes*
    b. *No*
    c. *Other*

13. In the protected area(s) where your primate-related work takes place and since the COVID-19 pandemic start in March 2020: visitor services or tourism facilities (visitor centers, camping sites, day use areas, trails, etc.) are:
    a. *Much better*
    b. *Somewhat better*
    c. *Stayed the same*
    d. *Somewhat worse*
    e. *Much worse*

14. In the protected area(s) where your primate-related work takes place and since the COVID-19 pandemic start in March 2020: conservation activities, such as patrolling, anti-poaching, monitoring, research, control of invasive species, and habitat restoration are:
    a. *Much better*
    b. *Somewhat better*
    c. *Stayed the same*
    d. *Somewhat worse*
    e. *Much worse*

15. In the protected area(s) where your primate-related work takes place and since the COVID-19 pandemic start in March 2020: public engagement, outreach and the provision of services to local communities in and around the protected area(s) is:
    a. *Much better*
    b. *Somewhat better*
    c. *Stayed the same*
    d. *Somewhat worse*
    e. *Much worse*

16. In the protected area(s) where your primate-related work takes place and since the COVID-19 pandemic start in March 2020: protected area staffing levels are:
    a. *Much better*
    b. *Somewhat better*
    c. *Stayed the same*
    d. *Somewhat worse*
    e. *Much worse*

17. In the protected area(s) where your primate-related work takes place and since the COVID-19 pandemic start in March 2020: working conditions, workloads, safety or well-being of protected area staff are:
    a. *Much better*
    b. *Somewhat better*
    c. *Stayed the same*
    d. *Somewhat worse*
    e. *Much worse*

18. In the protected area(s) where your primate-related work takes place and since the COVID-19 pandemic start in March 2020: financing of the protected area(s) is:
    a. *Much better*
    b. *Somewhat better*
    c. *Stayed the same*
    d. *Somewhat worse*
    e. *Much worse*

19. In your opinion, and since the COVID-19 pandemic started in March 2020, is primate conservation in the protected area(s) where you do your primate-related work:
    a. *Much better*
    b. *Somewhat better*
    c. *Stayed the same*
    d. *Somewhat worse*
    e. *Much worse*

20. Are there any particular measures that were introduced in protected areas in response to COVID-19 that will be continued after the pandemic is over?
    a. *Open response*

21. What lessons for protected areas can be learned from the COVID-19 pandemic? How should protected area management be changed in the post-COVID-19 era?
    a. *Open response*

Impact of COVID-19 on primate hunting:

22. Following the onset of the COVID-19 pandemic in March 2020, primates are hunted at the sites where you work:
    a. *Much more frequently*
    b. *More frequently*
    c. *At the same frequency as before*
    d. *Less frequently*
    e. *Much less frequently*
    f. *I do not know*
    g. *Other*

23. Why do you believe COVID-19 has changed or not changed the hunting of primates at the sites where you work? What have you observed (e.g. changes in hunting locations, hunting methods, etc.)?
    a. *Open response*

24. Do you know of any primates that have been killed specifically due to COVID-19? Please provide details if killings were due to, for example, hunger or fear that primates were carriers of COVID-19.
    b. *Open response*

25. Following the onset of the COVID-19 pandemic in March 2020, authorities enforce the law in regards to primate hunting in the sites/countries where you do your primate-related work:
    a. *Much better*
    b. *Somewhat better*
    c. *Stayed the same*
    d. *Somewhat worse*
    e. *Much worse*
    f. *I do not know*
    g. *Other*

26. Following the onset of the COVID-19 pandemic in March 2020, hunted/dead primates are appearing on social media:
    a. *Much more frequently*
    b. *More frequently*
    c. *At the same frequency as before*
    d. *Less frequently*
    e. *Much less frequently*
    f. *I do not know*
    g. *Other*

Impact of COVID-19 on primate pet trade (both international and within range countries):

27. How has the COVID-19 pandemic changed how often primates are kept as pets within the habitat range countries where you work?
    c. *Much more frequently*
    d. *More frequently*
    e. *At the same frequency as before*
    f. *Less frequently*
    g. *Much less frequently*
    h. *I do not know*
    i. *Other*

28. How has the COVID-19 pandemic changed the wellbeing of primates kept as pets within the habitat range countries where you work?
    j. *Much better*
    k. *Somewhat better*
    l. *Stayed the same*
    m. *Somewhat worse*
    n. *Much worse*
    o. *I do not know*
    p. *Other*

29. In the regions where you do your primate-related work, do you know of any pet primates that were released into the wild, sold, killed, or given away as a gift due to COVID-19?
    a. *Yes*
    b. *No*
    c. *I do not know*
    d. *Other*

30. Following the onset of the COVID-19 pandemic in March 2020, authorities enforce the law in regards to pet primate ownership in the sites/countries where you do your primate-related work:
    q. *Much better*
    r. *Somewhat better*
    s. *Stayed the same*
    t. *Somewhat worse*
    u. *Much worse*
    v. *I do not know*
    w. *Other*

31. In the regions/countries where you do your primate-related work, how has COVID-19 changed how and whether pet primates are appearing on social media?
    a. *Much more frequently*
    b. *More frequently*
    c. *At the same frequency as before*

d. Less frequently
e. Much less frequently
f. I do not know
g. Other

Closing questions:

32. Are there are any discussions underway in your organization to prepare for future outbreaks of infectious diseases, or similar kinds of disruption?
    a. Yes
    b. No
    c. Other

33. Is there anything else you would like to tell us as it relates to the impacts of COVID-19 on your primate research and/or conservation work?
    a. *Open response*

34. Please provide your email address if you would like to be sent the results of our survey.
    a. *Open response*
